# Acute Hyperbaric Oxygenation, Contrary to Intermittent Hyperbaric Oxygenation, Adversely Affects Vasorelaxation in Healthy Sprague-Dawley Rats due to Increased Oxidative Stress

**DOI:** 10.1155/2018/7406027

**Published:** 2018-04-29

**Authors:** Zrinka Mihaljević, Anita Matić, Ana Stupin, Lidija Rašić, Ivana Jukić, Ines Drenjančević

**Affiliations:** Department of Physiology and Immunology, Faculty of Medicine, Josip Juraj Strossmayer University of Osijek, Cara Hadrijana 10E, 31 000 Osijek, Croatia

## Abstract

The present study was aimed at assessing endothelium-dependent vasorelaxation, at measuring superoxide production in the aorta and femoral artery, and at determining antioxidative enzyme expression and activity in aortas of male Sprague-Dawley rats (*N* = 135), randomized to an A-HBO_2_ group exposed to a single hyperbaric oxygenation session (120′ of 100% O_2_ at 2.0 bars), a 24H-HBO_2_ group (single session, examined 24 h after exposure), a 4D-HBO_2_ group (4 consecutive days of single sessions), and a CTRL group (untreated group). Vasorelaxation of aortic rings in response to acetylcholine (AChIR) and to reduced pO_2_ (HIR) was tested in vitro in the absence/presence of NOS inhibitor L-NAME and superoxide scavenger TEMPOL. eNOS, iNOS, antioxidative enzyme, and NADPH oxidase mRNA expression was assessed by qPCR. Serum oxidative stress markers and enzyme activity were assessed by spectrometry, and superoxide production was determined by DHE fluorescence. Impaired AChIR and HIR in the A-HBO_2_ group were restored by TEMPOL. L-NAME inhibited AChIR in all groups. Serum oxidative stress and superoxide production were increased in the A-HBO_2_ group compared to all other groups. The mRNA expression of iNOS was decreased in the A-HBO_2_ and 24H-HBO_2_ groups while SOD1 and 3 and NADPH oxidase were increased in the 4D-HBO_2_ group. The expression and activity of catalase and glutathione peroxidase were increased in the 4D-HBO_2_ group as well. AChIR was NO dependent. Acute HBO_2_ transiently impaired vasorelaxation due to increased oxidative stress. Vasorelaxation was restored and oxidative stress was normalized 24 h after the treatment.

## 1. Introduction

Hyperbaric oxygenation (HBO_2_) is commonly used to improve injuries related to hypoxia and ischemia, including infections, such as meningococcal sepsis and gaseous gangrene [1, 2], myocardial infarction [3], cerebral ischemia [4], and even some neurodegenerative disorders [5–7]. Experimental [8] and clinical data suggest that intermittent HBO_2_ [9] decreases tissue edema, increases nitric oxide (NO) synthesis, changes vascular reactivity to stimuli [10], and inhibits neuroinflammatory factors' expression and apoptotic pathways [11].

Oxygen is a highly reactive molecule which, at high partial pressures (like in HBO_2_), can contribute to the increased formation of reactive oxygen species (ROS) [12] and affect system hemodynamics and vascular function. However, emerging data suggest that whether the ROS would be produced depends on hyperbaric protocol [13–18]. For example, our previous study showed that acute exposure to HBO_2_ increased plasma oxidative stress (measured by lipid peroxidation products), decreased systolic and diastolic blood pressure, decreased pH and pCO_2_ in arterial blood, and increased pO_2_ in healthy SD rats [19]. On the other hand, intermittent HBO_2_ restored vascular relaxation [20] and changed the metabolic pathways involved in the vasorelaxation in healthy and diabetic animals. However, it did not affect oxidative stress markers which were persistently increased in DM rats [10]. Taken together, HBO_2_ may influence functional and structural characteristics of blood vessels, depending on the application protocol.

Antioxidant systems counter-effect the damage induced by ROS. That includes antioxidative enzymes, such as glutathione peroxidase, superoxide dismutase, or catalase [21]. On the other side, there is the nonenzymatic intracellular and extracellular antioxidant defense system [22] which includes different chemical groups, for example, vitamins, carotenoids, amino acids, and peptides, established in various cellular structures. All antioxidant factors of the body, either intracellular enzymes or antioxidant compounds (nonenzymatic factors), are called total antioxidant capacity. In a study conducted by Winston et al., it was revealed that glutathione, ascorbic acid (vitamin C), uric acid, and vitamin E compose 70% of the total antioxidant capacity of the body [23]. At the moment, only few studies examined the effect of HBO_2_ treatment on antioxidative defense capacity [21, 24–26], which may be an important modulator of diverse effects of acute and chronic or intermittent hyperoxygenation. We have found that plasma antioxidant capacity has not been affected by intermittent HBO_2_ protocol in diabetic rats [10].

Thus, the purpose of this study was to test the hypothesis that acute HBO_2_ leads to increased superoxide production, which underlies impaired endothelium-dependent vasorelaxation, in contrast to the effects of repeated exposures to HBO_2_ (intermittent HBO_2_), which is beneficial for the vasorelaxation in healthy rats. The experiments conducted in the present study were designed to (1) test the effect of HBO_2_ on vascular endothelial-dependent reactivity in healthy rats exposed to acute (A-HBO_2_), 24 h after a single exposure (24H-HBO_2_) and intermittent HBO_2_ (4D-HBO_2_) compared to untreated healthy rats (CTRL) in regard to oxidative stress; (2) assess in situ aortic and femoral artery superoxide production of rats exposed to hyperbaric oxygenation; and (3) determine the expression of antioxidative enzymes in rat aortic tissue after various hyperbaric oxygenation protocols.

## 2. Materials and Methods

### 2.1. Experimental Animals

The animals were bred and housed at the animal care facility of the Faculty of Medicine Osijek. All experimental procedures conformed to the European Guidelines for the Care and Use of Laboratory Animals (directive 86/609) and were approved by the local and national Ethical Committee (no. 2158/61-02-139/2-06).

A total of 135 male Sprague-Dawley (SD) rats (age 9–12 weeks) were used in this study. Rats were housed in a temperature- (21°C–23°C), humidity-, and light-controlled room with free access to tap water and fed ad libitum with a commercially prepared pellet diet (Mucedola, Italy).

### 2.2. Hyperbaric Oxygen (HBO_2_) Treatment

Hyperbaric groups underwent single (A-HBO_2_ and 24H-HBO_2_ groups) or four (one per day, intermittent, 4D-HBO_2_) 120-minute sessions of 100% O_2_ at 2.0 bars absolute of pressure with additionally 15 minutes for gradual compression and decompression (Recompression Chamber for Experiments 110L, Djuro Djakovic, Aparati d.d., Slavonski Brod, Croatia).

### 2.3. Blood Pressure Measurements: Surgical Procedure and Sample Collection

Invasive blood pressure measurement was done by a procedure described by Drenjancevic et al. [19]. Shortly, the left femoral artery was cannullated in anaesthetized rat. Body temperature was maintained constant during the measurement, and blood pressure was monitored with a Spacelabs Medical monitor system (Spacelabs Medical Inc., Redmond, WA, USA). The systolic and diastolic blood pressure was determined after 10 minutes of stabilization as average blood pressure recorded within one minute of measurement every 10 seconds. The mean arterial pressure (MAP) was calculated as the sum of the systolic blood pressure and double diastolic blood pressure divided by 3. The same rats were used to collect aortas and arterial blood serum samples for further analysis of oxidative stress and qPCR.

### 2.4. Measurement of Isometric Tension of Isolated Rat Aortic Rings

The aortic ring experiments were done according to the protocol already described in our laboratory [8, 10, 27]. On the day of the experiment and immediately before decapitation, all rats were anaesthetized with 75 mg/kg of ketamine (Ketanest S 25 mg/ml, Pfizer) and 0.5 mg/kg of midazolam (Midazolam Torrex 5 mg/ml, Torrex Chiesi Pharma). After decapitation, the descending thoracic aorta was dissected free from the connective tissue, placed in oxygenated modified Krebs-Henseleit solution, and cut into rings of about 3–4 mm in length. The solution consisting of (mM) NaCl 113, KCl 4.7, MgSO_4_·6H_2_O 1.2, NaHCO_3_ 22.0, CaCl_2_·2H_2_O 1.3, KH_2_PO_4_ 1.2, EDTA 0.026, and glucose 11.1 was bubbled with a gas mixture of 95% O_2_ and 5% CO_2_ throughout the experiment, and was kept at a temperature of 37°C. Two stainless-steel hooks were inserted into the lumen of each aortic ring and mounted on a holder that was further placed in a 10 ml organ bath, and the upper wire was connected to the transducer's arm via filament. The WPI Inc. system and PowerLab data acquisition and analysis software were used. Basal resting tension of 2.0 g was applied on each aortic ring and allowed to equilibrate for 60 minutes, replacing the Krebs–Henseleit solution every 15 min with fresh solution and readjusting passive tension to 2.0 g as needed. Subsequently, the intactness of the endothelium was tested by precontracting the rings with 10^−7^ M (final concentration) noradrenaline, letting to stabilize for 5 min and inducing relaxation with 10^−5^ M acetylcholine. If the vessel ring failed to relax, it was not used for further studies. If the vessel ring relaxed, it was washed three times with fresh solution and allowed to equilibrate for 30 min, with washing at 10 min intervals. After the rings were stabilized, maximal contraction was induced with 60 mM KCl + 10^−7^ M noradrenaline. When plateau was reached, the rings were washed three times with fresh solution and allowed to equilibrate for 30 min, washing at 10 min intervals. After this phase, aortic ring responses to ACh or hypoxia were done.

### 2.5. Protocols for Functional Vascular Studies in Aortic Rings

After triple washout and tension stabilization, the rings from all groups were subjected to one of the following experimental protocols. To assess baseline vasorelaxing potential, the intact rings from all groups were exposed to cumulative concentrations of ACh (acetylcholine chloride, Sigma-Aldrich, USA) (10^−9^–10^−5^ M). To evaluate the role of NO and oxidative stress in the ACh-induced vasorelaxation, the intact rings from all groups were incubated for 10 min with (a) a NO synthase inhibitor, L-nitro-arginine methyl ester (L-NAME) (L-NAME hydrochloride, 1 g, Sigma, USA) (3 × 10^−4^ M), to determine the role of NO in vascular relaxation and (b) a SOD mimetic, TEMPOL (4-hydroxy-2,2,6,6-tetramethylpiperidine-1-oxyl, 25 g, Acros Organics, USA) (100 *μ*M), which was used before exposure to cumulative concentrations of ACh (10^−9^–10^−5^ M), to evaluate the role of superoxide.

After the equilibration and recovery period, rings were precontracted with NE (10^−7^ M), and the gas mixture was switched from 95% O_2_ and 5% CO_2_ to 0% O_2_ with 5% CO_2_ for 20 minutes and then switched back to 95% O_2_ with 5% CO_2_ for 5 minutes for reoxygenation. To evaluate the role of oxidative stress on the hypoxia-induced vasodilation, rings from all groups were incubated for 10 minutes with TEMPOL (100 *μ*M final concentration) and hypoxia protocol was repeated. The relaxation was expressed as the percentage decrease of the NE-induced vasoconstriction.

The sensitivity of the smooth vascular muscle to NO was tested on intact rings from all groups. The aortic rings were exposed to cumulative doses of SNP (sodium nitroprusside dihydrate, 10^−10^ to 10^−4^ M), an endothelium-independent NO donor.

### 2.6. Serum Oxidative Stress Parameters' Analysis

Thiobarbituric acid-reactive substance (TBARS) assay and plasma antioxidant capacity (FRAP) assay were performed according to the established protocol in our laboratory [27, 28]. Arterial blood samples were collected from the femoral artery and centrifuged on 3500 rpm for 10 minutes, and serum samples were stored at −80°C until use. TBARS assay is based on the reaction of malondialdehyde (MDA), an end-product of lipid peroxidation, with TBARS. To correct for background absorption, the absorbance values at 572 nm were subtracted from those at 532 nm, which represent the absorption maximum of the TBA:MDA adduct [29]. Absorbance was monitored by NanoPhotometer® P-Class P330-30 (Implen, Germany). Results were compared with a standard curve with MDA and expressed as *μ*M MDA equivalents. The antioxidant capacity of plasma was measured by the ferric reducing antioxidant power (FRAP) assay. In this assay, antioxidants are evaluated as reductants of Fe^3+^ to Fe^2+^, which is chelated by TPTZ (2,4,6-tris(2-pyridyl)-s-triazine) to form a Fe^2+^–TPTZ complex absorbing at 593 nm [22]. Results were compared with a standard curve with Trolox (TE), a water-soluble analogue of vitamin E, and expressed as *μ*M TE equivalents.

### 2.7. In Situ Evaluation of O_2_^−^· Levels with Dihydroethidine (DHE) by Fluorescence Microscopy

Evaluation of O_2_^−^· levels was performed according to the protocol described by Zhu et al. [30]. Aortic ring samples were prepared as for functional vascular studies and cut into four rings of about 2–3 mm in length, precalibrated, and equilibrated for one hour at 37°C in Krebs–Henseleit solution and bubbled with a gas mixture of 95% O_2_ and 5% CO_2_. After the equilibration period, to assess the influence of TEMPOL on O_2_^−^· levels, 2 rings were incubated for 30 minutes in the same dosage as for functional studies (100 *μ*M final concentration). To assess the NOS_3_ uncoupling effect, we performed an incubation with L-NAME (3 × 10^−4^ M) (only on aortic rings). After the incubation period, aortic rings were transferred to HEPES buffer (137 mM NaCl, 5.4 mM KCl, 4.2 mM NaHCO_3_, 3 mM Na_2_HPO_4_, 0.4 mM KH_2_PO_4_, 0.5 mM MgCl_2_·6H_2_O, 0.8 mM MgSO_4_·7H_2_O, 10 mM glucose, 20 mM HEPES, and 1.2 mM CaCl_2_·H_2_O) containing specific dye. The femoral artery about 3–4 mm in length was isolated and prepared in the same manner.

To assess the production of superoxide radicals, the aortic rings and femoral arteries were loaded with dihydroethidine (DHE, 20 *μ*M) for 45 min in HEPES solution at 37°C. DHE enters the cell, and ethidium binds to DNA in the cell, resulting in a strong red fluorescence. Measurements of DHE fluorescence were performed on a Zeiss Axioskop MOT2 microscope with an Olympus DP70 camera using a Zeiss filter set 15 with a 546 nm wavelength for excitation and a 590 nm wavelength for emission with a beam splitter at 580 nm. Images were processed and analyzed by ImageJ software following the software developer's instructions (National Institutes of Health) [31, 32].

### 2.8. mRNA Expression of Antioxidative Enzymes in Rat Aortas

Rat aorta samples were isolated, immediately frozen in liquid nitrogen, and stored at −80°C until RNA isolation. Homogenization of samples and total RNA was extracted using TRI reagent (Life Technologies, USA) according to protocol by Chomczynski et al. (1987) [33], also established in our laboratory [28]. RNA purity and concentration were assessed by NanoPhotometer P-Class P330-30 (Implen, Germany). RNA was purified using deoxyribonuclease I kit (Sigma, USA), and cDNA was synthesized by the High Capacity cDNA kit with RNase Inhibitor (Applied Biosystems, USA). Quantitative real-time PCR was performed on the CFX96 system (Bio-Rad, USA) to assess the relative expression of eNOS, iNOS, superoxide dismutase isoforms 1, 2, and 3 (Cu/Zn SOD, Mn SOD, EC SOD → SOD 1, 2, and 3), catalase, glutathione peroxidase 1 and 4 (GPx1 and GPx4), and nicotinamide adenine dinucleotide phosphate (NADPH) oxidase components (p47phox and gp91phox). Gene expression was normalized to the HPRT (hypoxanthine-guanine phosphoribosyltransferase) gene (HPRT expression in Table 1). Expression of these genes was determined using ABsolute qPCR SYBR Green Low ROX Mix (Thermo Scientific, Lithuania). All experiments were performed at the Dept. of Physiology and Immunology, Laboratory for Physiology of Circulation, and Laboratory for Molecular and Clinical Immunology and the Dept of Medical Biology and Genetics (fluorescence measurements), Faculty of Medicine, University of Osijek.

### 2.9. Spectrophotometric Antioxidant Enzyme Activity Assay

Spectrophotometric antioxidant enzyme activities were measured according to the protocol described by Ćosić et al. [28]. Fresh aorta samples were weighed and pulverized first with liquid nitrogen into which buffer is then added (100 mM phosphate buffer + 1 mM EDTA, pH 7.0) in proportion to the quantity-weighed tissue (1 ml buffer per 100 mg tissue) and then additionally homogenized with Ultra Turrax T10 homogenizer (1300 rpm; IKA, Königswinter, Germany). Tissue homogenates were then centrifuged at 20000*g* for 15 minutes at 4°C, and supernatants were stored at −80°C until assayed.

Catalase (CAT) activity was measured according to protocol by Aebi [34], using 0.036% hydrogen peroxide (H_2_O_2_) as a substrate in the reaction mixture with 50 mM phosphate buffer pH 7.0. Changes in absorbance in the reaction mixture were measured at 240 nm during 2 minutes every 10 seconds after adding the sample. One unit of activity corresponds to the loss of 1 *μ*mol of H_2_O_2_ per minute.

Glutathione peroxidase (GPx) activity [35] was determined indirectly by measuring the rate of nicotinamide adenine dinucleotide phosphate (NADPH) oxidation to NADP^+^, accompanied by a decrease in absorbance at 340 nm during 5 minutes. In that assay, one unit of GPx activity catalyzes the oxidation by H_2_O_2_ of 1.0 *μ*mol of reduced glutathione to oxidized glutathione per minute at pH 7.0 and 25°C.

Total SOD activity in the supernatant is determined by the ability to inhibit the reduction of cytochrome C by the superoxide anion by the addition of xanthine and xanthine oxidase. Activity was measured according to a modified method described by Flohé and Ötting [36]. Calibrations were performed with the use of a known amount of purified bovine SOD.

Measured activities of all investigated enzymes were expressed as units of the enzymes per milligram of protein (U/mg protein). Enzyme activity assay was performed using a Lambda 25 UV-Vis spectrophotometer equipped with UV WinLab 6.0 software package (PerkinElmer For the Better, Waltham, Massachusetts, USA). The concentration of proteins in samples (mg/ml) was determined following the protocol from the manufacturer of Bradford reagent at 595 nm (Bradford Reagent B6916, Sigma-Aldrich), using bovine serum albumin as a standard.

### 2.10. Statistical Analysis

All data are expressed as means ± standard error of the means (SEM). *p* < 0.05 was considered statistically significant. Data were analyzed using GraphPad Prism version 5.0 for Windows and SigmaPlot (version 11.2, Systat Software Inc., Chicago, USA). The normality of data distribution was assessed by the Kolmogorov–Smirnov normality test, and in the case of unequal distribution, nonparametric tests were applied. The data obtained from functional studies were analyzed by two-way ANOVA followed by the Bonferroni post hoc test for both pairwise comparisons and comparisons versus a control group. The analysis of oxidative stress and antioxidant capacity was performed using one-way ANOVA or Kruskal-Wallis test followed by the Holm-Sidak post hoc test, respectively. One-way ANOVA or the Kruskal-Wallis test was used for data analysis of real-time PCR where appropriate (Systat Software Inc., USA).

## 3. Results

### 3.1. General Information and Blood Pressure of Studied Groups

Body mass (g) of rats was not significantly different among examined groups. Data on the systolic, diastolic, and mean arterial pressure before (CTRL) and immediately after HBO_2_ (A-HBO_2_), 24 hours after HBO_2_ (24H-HBO_2_ group), and 4 days after HBO_2_ (4D-HBO_2_ group) are listed in Table 2. There was a significant decrease in systolic and diastolic blood pressure in the A-HBO_2_ group compared to the control group. No changes in these variables were observed in other groups.

### 3.2. Acethylcholine-Induced Vasorelaxation (Endothelium-Dependent) of Isolated Rat Aortic Rings

**Figure 1:**
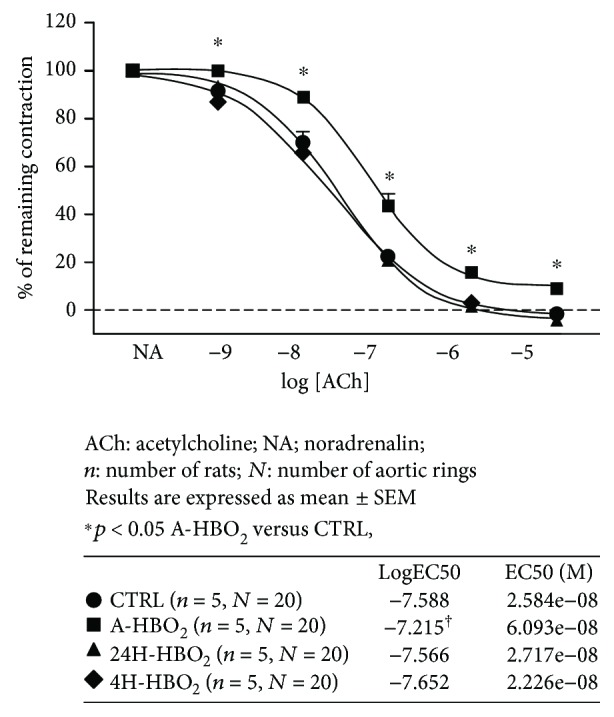
ACh-induced relaxation (AChIR) of isolated rat aorta rings in the CTRL, A-HBO_2_, 24H-HBO_2_, and 4D-HBO_2_ groups. AChIR was significantly impaired in the A-HBO_2_ group when compared to the other groups of rats with a 10^−9^–10^−5^ M ACh concentration. A-HBO_2_ rats exhibited lower sensitivity to ACh compared to other groups of rats (table). Half maximal effective concentration (EC50) presents concentration of ACh (M) which induces a response halfway between the baseline and maximum. LogEC50 values (shown in the corresponding tables) were compared by a one-way ANOVA test.

Figure 1 presents the results of isolated aortic ring vasorelaxation in response to ACh (AChIR) in all experimental groups of rats. AChIR was significantly reduced in the A-HBO_2_ group compared to all other groups of animals. A-HBO_2_ rats also exhibited lower sensitivity to ACh compared to other groups of rats (presented by logEC50 in the tables with each graph).

**Figure 2:**
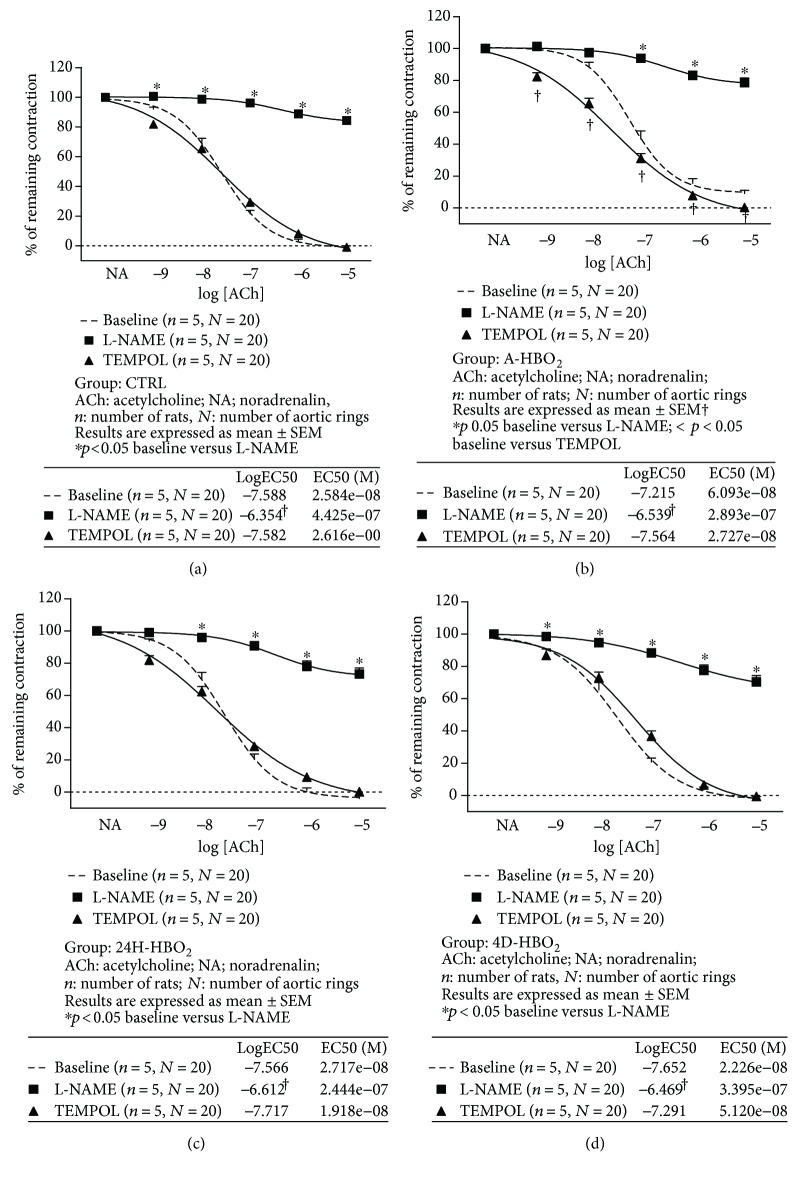
Mechanisms of AChIR response of isolated rat aorta rings in the CTRL (a), A-HBO_2_ (b), 24H-HBO_2_ (c), and 4D-HBO_2_ (d) groups of rats. Used concentrations: ACh 10^−9^ to 10^−5^ M, L-NAME 3 × 10^−4^ M, and TEMPOL 100 *μ*M. Half maximal effective concentration (EC50) presents the concentration of ACh (M) which induces a response halfway between the baseline and maximum. The presence of L-NAME significantly reduced AChIR of isolated rat aortic rings in all experimental groups. TEMPOL administration significantly increased the AChIR response in the A-HBO_2_ group of rats, while it did not induce any significant change in the AChIR of isolated rat aortic rings in other groups. Data were compared by two-way ANOVA and Bonferroni post hoc tests. Tables within figures present sensitivity of aortic rings to ACh in the CTRL (a), A-HBO_2_ (b), 24H-HBO_2_ (c), and 4D-HBO_2_ (d) groups of rats. Sensitivity to ACh in the presence of L-NAME was significantly decreased compared to the basic response or response to ACh in the presence of TEMPOL in all experimental groups of rats. LogEC50 values were compared by one-way ANOVA followed by a Holm-Sidak pairwise multiple comparison.

**Figure 3:**
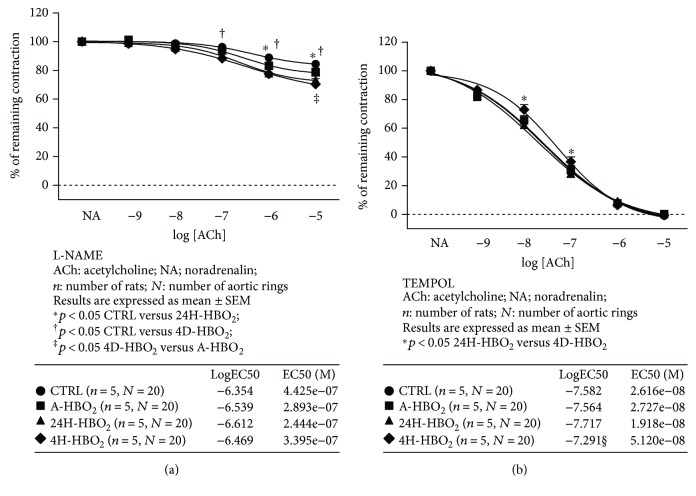
Relaxation to acetylcholine in the presence of eNOS inhibitor L-NAME (a) and superoxide scavenger TEMPOL (b) in the CTRL, A-HBO_2_, 24H-HBO_2_, and 4D-HBO_2_ groups of rats. The presence of L-NAME inhibited AChIR more prominently in CTRL compared to 24H-HBO_2_ and 4D-HBO_2_ groups, and in A-HBO_2_ that in the 4D-HBO_2_ group of rats. AChIR in the presence of TEMPOL was lower in 4D-HBO_2_ than in the 24H-HBO_2_ group for ACh concentrations 10^−8^ and 10^−7^ M. Data were compared by two-way ANOVA and Bonferroni post hoc tests. Tables within figures present sensitivity of aortic rings to ACh in the presence of L-NAME (a) and TEMPOL (b) in the CTRL, A-HBO_2_, 24H-HBO_2_, and 4D-HBO_2_ groups of rats. Sensitivity to ACh in the presence of L-NAME did not differ among experimental groups of rats. Sensitivity to ACh in the presence of TEMPOL was significantly decreased in the 4D-HBO_2_ group compared to the other groups of rats. LogEC50 values were compared by one-way ANOVA followed by a Holm-Sidak pairwise multiple comparison.

The role of NO and oxidative stress in AChIR is presented in Figure 2. In all experimental groups of rats, ACh relaxation was mediated mainly by NO and was significantly inhibited by the addition of eNOS inhibitor L-NAME. NO contributed less to AChIR in the 24H-HBO_2_ group (at ACh 10^−6^ and 10^−5^ M) and in the 4D-HBO_2_ group (at ACh 10^−7^, 10^−6^, and 10^−5^ M) compared to the untreated controls (Figure 3(a)). Still, contribution of NO to AChIR was less in the 4D-HBO_2_ group than in the A-HBO_2_ group of rats (Figure 3(a)). There were no differences in sensitivity to ACh in the presence of L-NAME among groups (logEC50 values in the tables within Figure 3(a)).

However, in all tested groups, within-group tests revealed that in the presence of L-NAME, the sensitivity to ACh was significantly lower compared to the basic response to ACh and response to ACh in the presence of TEMPOL in the corresponding group (logEC50 values in the tables within Figures 2(a)–2(d)).

In the A-HBO_2_ group, in vitro superoxide scavenger TEMPOL restored the relaxation response to ACh similar to the basal values of other experimental groups (Figure 2(b)). TEMPOL did not affect AChIR in the CTRL (Figure 2(a)), 24H-HBO_2_ (Figure 2(c)), and 4D-HBO_2_ groups (Figure 2(d)). In the 4D-HBO_2_ group, AChIR in the presence of TEMPOL was lower compared to 24H-HBO_2_ (at ACh 10^−8^ and 10^−7^ M) (Figure 3(b)). Sensitivity to ACh after TEMPOL incubation was also significantly decreased in 4D-HBO_2_ compared to the other groups.

### 3.3. Hypoxia-Induced Vasorelaxation (HIR) of Isolated Rat Aortic Rings

**Figure 4:**
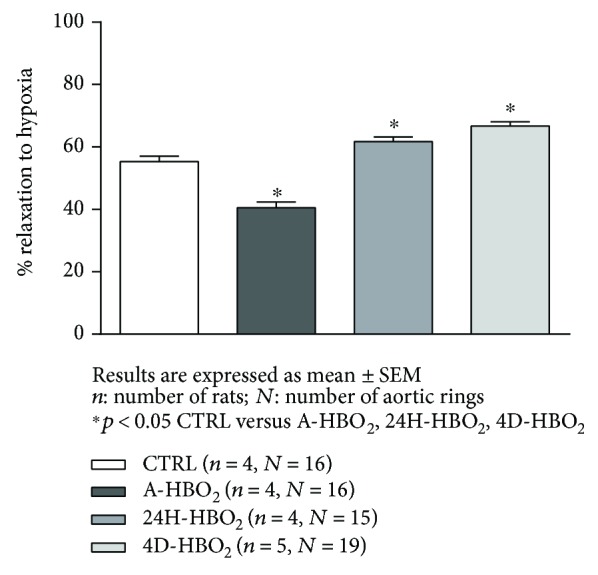
Hypoxia-induced relaxation (HIR) response of isolated rat aorta rings in the CTRL, A-HBO_2_, 24H-HBO_2_, and 4D-HBO_2_ groups. HIR was significantly lower in A-HBO_2_ compared to all other groups of rats. The 24-HBO_2_ and 4D-HBO_2_ groups exhibited significantly increased HIR compared to CTRL and A-HBO_2_ groups of rats.

A-HBO_2_ groups exhibited significantly decreased vasorelaxation in response to hypoxia compared to all other groups of rats. The 24H-HBO_2_ and 4D-HBO_2_ groups exhibited significantly enhanced vasorelaxation in response to hypoxia compared to the CTRL and A-HBO_2_ groups of rats (Figure 4).

**Figure 5:**
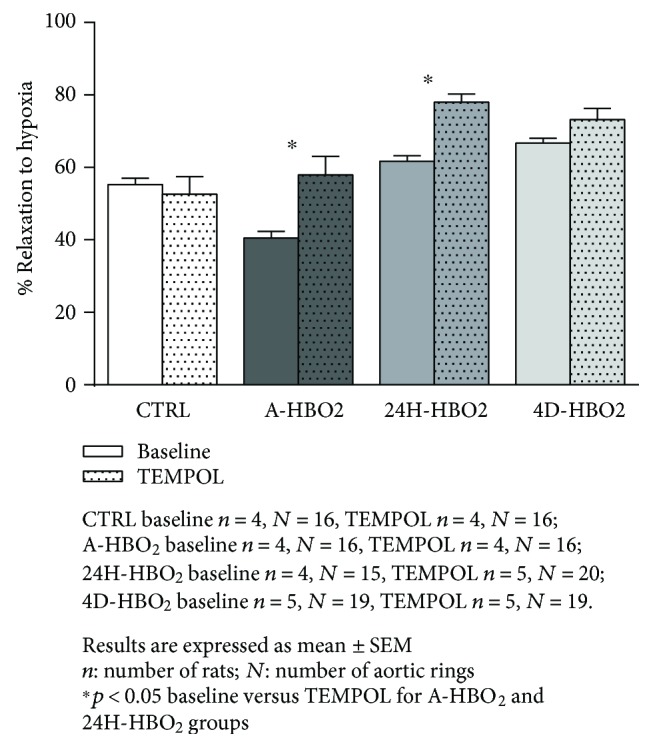
Relaxation to hypoxia in the presence of superoxide scavenger TEMPOL in the CTRL, A-HBO_2_, 24H-HBO_2_, and 4D-HBO_2_ groups of rats. TEMPOL administration significantly increased HIR compared to baseline measurement in the A-HBO_2_ and 24H-HBO_2_ groups of rats.

After incubation of aortic rings with TEMPOL, vasorelaxation in response to hypoxia in the A-HBO_2_ group was restored to the levels similar to the control group. There was no effect of TEMPOL on HIR in the CTRL group. However, in the presence of TEMPOL, HIR in the 24H-HBO_2_ group was significantly increased compared to baseline (Figure 5).

### 3.4. Sodium Nitroprusside-Induced Vasorelaxation (Endothelium-Independent) of Isolated Rat Aortic Rings

**Figure 6:**
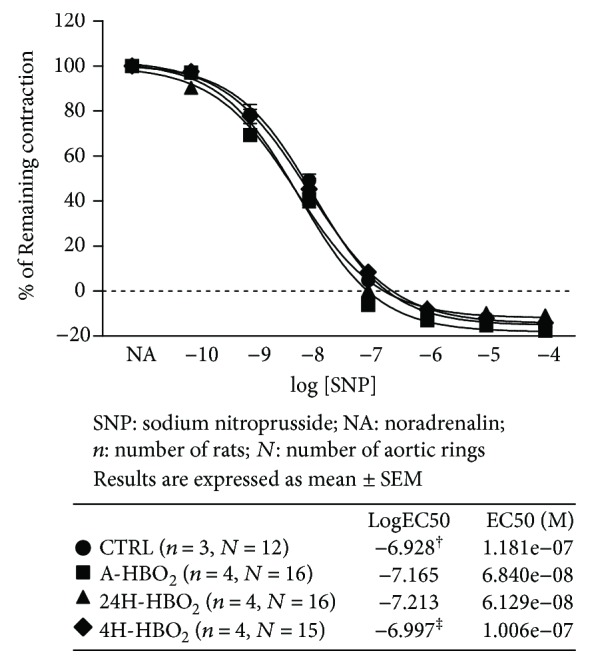
SNP-induced relaxation (SNPIR) of isolated rat aorta rings in the CTRL, A-HBO_2_, 24H-HBO_2_, and 4D-HBO_2_ groups. There was no difference in SNPIR among experimental groups of rats. Data were compared by two-way ANOVA and Bonferroni post hoc tests. The CTRL group exhibited lower sensitivity to SNP compared to the A-HBO_2_ and 24H-HBO_2_ groups of rats (^†^*p* < 0.05); the 4D-HBO_2_ group exhibited lower sensitivity to SNP compared to the 24H-HBO_2_ group of rats (^‡^*p* < 0.05) (table). Half maximal effective concentration (EC50) presents concentration of SNP (M) which induces a response halfway between the baseline and maximum. LogEC50 values (shown in corresponding tables) were compared by one-way ANOVA test.

Aortic ring relaxation in response to sodium nitroprusside (SNP), an endothelium-independent NO donor, was similar between all tested groups (Figure 6). A-HBO_2_ and 24H-HBO_2_ groups exhibited higher sensitivity to SNP compared to CTRL, and the 24H-HBO_2_ group exhibited higher sensitivity to SNP compared to 4D-HBO_2_ (logEC50 values in the table within the graphs in Figure 6).

### 3.5. Oxidative Stress, Antioxidative Capacity, and Superoxide Production

Serum TBARS, the measure of lipid oxidation products, was significantly increased in the A-HBO_2_ group compared to the CTRL, 24H-HBO_2_, and 4D-HBO_2_ groups. There was no significant difference in antioxidant capacity (FRAP values) among all tested groups (Table 3).

**Figure 7:**
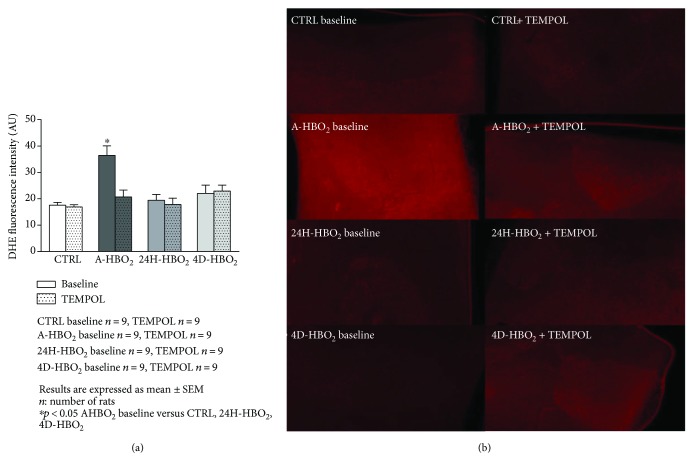
(a) DHE superoxide fluorescence intensity measurement in rat aorta. The increase in superoxide levels in the aortas of rats exposed to hyperbaric oxygen was prevented by incubation with TEMPOL. Data are shown as means ± SEM of DHE fluorescence intensity units. ^∗^Significant increase in superoxide levels compared with control value before any treatment (*p* < 0.05; *n* = 9 rats). (b) In situ fluorescence of superoxide after dying of aortic rings with DHE color in all tested groups before and after incubation with TEMPOL. The color intensity is proportional to the amount of superoxide. The images were made on the Zeiss Axioskop MOT2 microscope, using the Olympus DP70 camera with the Zeiss filter set 15 (546 nm wavelength for excitation and 590 nm for emission with a beam splitter at 580 nm).

**Figure 8:**
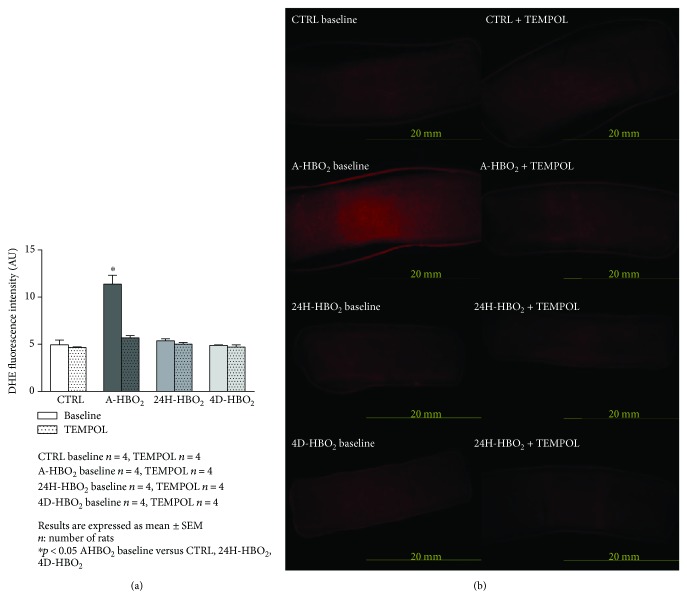
(a) DHE superoxide fluorescence intensity measurement in the rat femoral artery. The increase in superoxide levels in the artery of rats exposed to a single session of hyperbaric oxygen was prevented by incubation with TEMPOL. Data are shown as means ± SEM of DHE fluorescence intensity units. ^∗^Significant increase in superoxide levels compared with control value before any treatment (*p* < 0.05; *n* = 4 rats). (b) In situ fluorescence of superoxide after dying of the femoral artery with DHE color in all tested groups before and after incubation with TEMPOL. The color intensity is proportional to the amount of superoxide. The images were made on the Zeiss Axioskop MOT2 microscope, using the Olympus DP70 camera with the Zeiss filter set 15 (546 nm wavelength for excitation and 590 nm for emission with a beam splitter at 580 nm).

**Figure 9:**
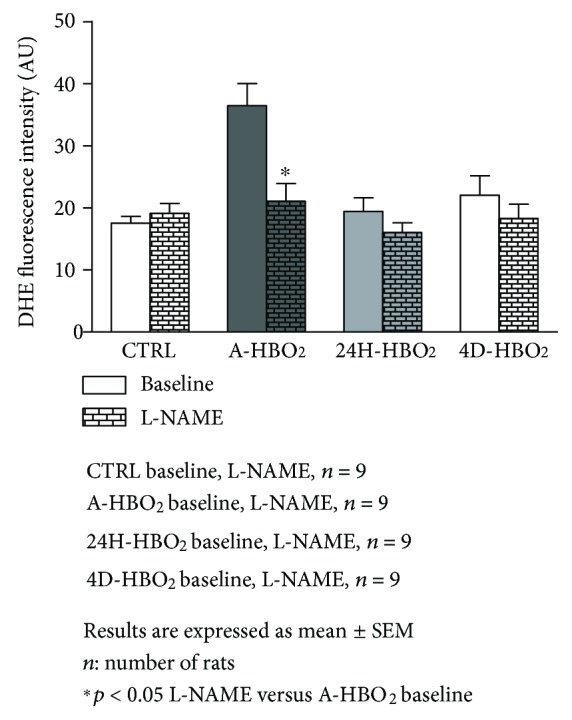
DHE superoxide fluorescence intensity measurement in the rat aorta before and after incubation with L-NAME. The superoxide levels in the aortas of rats exposed to a single session of hyperbaric oxygen was decreased by incubation with L-NAME compared to baseline. Data are shown as means ± SEM of DHE fluorescence intensity units. ^∗^Significant decrease in superoxide levels after L-NAME incubation compared with baseline value (*p* < 0.05; *n* = 9 rats).

Aortic and femoral artery superoxide production was significantly increased in the A-HBO_2_ group compared to all other groups. TEMPOL in vitro significantly decreased superoxide production in the A-HBO_2_ group similar to basal values of other groups (Figures 7(a), 7(b), 8(a), and 8(b)). L-NAME in vitro significantly decreased superoxide production in the A-HBO_2_ group in aortic rings and showed no effect on other groups (Figure 9).

### 3.6. mRNA Expression of Oxidative and Antioxidative Genes in the Rat Aortic Tissue

Aortic SOD1 and SOD3 gene mRNA expression was significantly higher in the 4D-HBO_2_ group compared to all other groups. Aortic mRNA expression of SOD2 was not significantly different among groups. Expression of catalase, GPx1, GPx4, and NADPH oxidase components was significantly increased in the 4D-HBO_2_ group compared to the control group, while expression of GPx1 and NADPH oxidase components mRNA was also significantly increased in the 4D-HBO_2_ group compared to A-HBO_2_ and 24H-HBO_2_. Catalase and GPx4 mRNA expression was significantly increased in the 24H-HBO_2_ group compared to the control group (Table 4).

The relative aortic mRNA expression of iNOS was significantly decreased in the A-HBO_2_ and 24H-HBO_2_ groups compared to the control group, while expression of eNOS was not significantly different among groups (Table 4). The relative expression of mRNA of studied genes was normalized to the HPRT (hypoxanthine-guanine phosphoribosyltransferase) gene mRNA expression (HPRT expression in Table 1).

### 3.7. Antioxidant Enzyme Activities

Activity of catalase in aortic tissue is significantly increased in the 4D-HBO_2_ group compared to the CTRL and A-HBO_2_ groups, and activity of GpX is increased in the 4D-HBO_2_ group compared to all other groups. There are no differences in SOD activity among tested groups (Table 5).

## 4. Discussion

The main findings of the present study are as follows: acute single exposure to HBO_2_ impairs vasorelaxation in response to ACh and hypoxia. These impaired responses are restored by superoxide scavenging. Superoxide production was increased in acute hyperbaric treatment and not affected by intermittent hyperbaric treatment. In addition, antioxidative enzyme expression, as well as iNOS expression, was increased in intermittent hyperbaric treatment. These results suggest that impaired vasorelaxation to ACh and hypoxia in acute HBO_2_ may be influenced by increased vascular oxidative stress, that is, superoxide formation. Increased oxidative stress is supported by findings of increased serum lipid peroxidation products and increased aortic superoxide production in the acute single exposure group (A-HBO_2_). Hink et al. [14] also reported that acute HBO_2_ exposure has decreased ACh relaxation, but they did not show a scavenging effect on superoxide and hydrogen peroxide by PEG-SOD and PEG-catalase and therefore could not explain such decrease. They performed in vitro experiments simulating HBO_2_ conditions. It is therefore possible that an increase in PO_2_ in buffer shortens the lifetime and, hence, decreases the bioavailability of NO which then leads to a decrease in Ach-induced relaxation. In contrast, we exposed living animals in a hyperbaric chamber and isolated the vessels after decompression and then observed the decrease in ACh-induced relaxation. Luo et al. [37] showed an almost equally scavenging effect of PEG-SOD and TEMPOL, and therefore, improvement of vascular relaxation in our experiment can be connected to superoxide scavenging. The present study is the first one to directly assess the production of superoxide and expression of antioxidative enzymes in different HBO_2_ protocols and to relate it to vascular reactivity.

In the present study, NOS_3_ uncoupling could be a source of increased superoxide production immediately after HBO_2_ treatment. Active NOS_3_, which is the dominant isoform in the endothelium [38, 39], is a homodimer that generates NO and L-citrulline from L-arginine. When exposed to oxidative stress or deprived of its reducing cofactor tetrahydrobiopterin (BH4) or substrate L-arginine, NOS_3_ uncouples to the monomeric form which generates O_2_^−^· rather than NO [40–42]. Uncoupled NOS_3_ is thought to be a prominent source of endothelial ROS in various disorders connected to endothelial dysfunction, such as hypertension [43]. However, in long-term exposure, the main source of superoxide seems to be NADPH. We observed an increased mRNA expression of NADPH oxidase components in the 4D-HBO_2_ group, and our experiments with L-NAME have not demonstrated a decrease in superoxide production in that group (Figure 9).

Endothelial oxygen-derived free radicals can inactivate NO formation [44, 45]; therefore, the effect of acute HBO_2_ exposure on ACh-induced relaxation, found to be reversible, is consistent with the study by Ay et al., who showed that the oxidative effect of HBO_2_ persists only for 1 h [46]. The present study also demonstrates that the effects of a hyperbaric oxygenation in producing increased oxidative stress and impaired endothelium-dependent vasorelaxation are extremely rapid, but transient, because significant changes were detected after only one exposure to HBO_2_, but have been lost 24 h after exposure (Figures 1 and 4, Table 3). Results of the present study suggest reduced contribution of NO to ACh-induced relaxation in HBO_2_-exposed animals, despite increased sensitivity of the vascular smooth muscle to NO, which seems to be in proportion to the duration of HBO_2_ treatment. Namely, HBO_2_ exposure can provoke adoptive mechanisms and alleviate oxidative stress even in healthy animals (Figure 3(a)). In concordance to these findings are recent studies from our laboratory presented by Kibel et al. [8] and Unfirer et al. [10] which showed the presence of an alternative pathway(s) of endothelium-dependent vasorelaxation to acetylcholine and ANG (1–7) in diabetic animals exposed to 4 days of hyperbaric oxygen, most probably involving enhanced production or sensitivity to EETs (epoxyeicosatrienoic acid). Studies in animal models show that there is an interaction between the enzymes that produce the vasoactive metabolites (NOS, COX-1, COX-2, and CYP450) and oxygen-free radicals (ROS) [47]. The ROS may be by-products of impaired activation of these enzymes together with the activation of NAD(P)H oxidase, while ROS may act on COX enzymes in a manner that changes its direction in the production of vasoconstrictor metabolites. In addition, ROS may act by reducing the bioavailability of NO [47].

Previous studies of hyperbaric oxygen exposure showed increased oxidative stress after long-term exposure [13, 24–26]. This is in contrast to our previous [10] and present study, mainly by the duration and manner of exposure to HBO_2_, as well as the time of tissue harvesting. In addition, none of the mentioned studies examined the effect of HBO_2_ on antioxidant enzyme expression [13, 24–26]. Previously, we hypothesized [19, 48] that the time between two exposures can be observed as pseudohypoxia which is important for upregulating antioxidative enzymes (as demonstrated in Table 4), as well as other enzymes important for maintaining vascular relaxation mechanisms [19, 48]. In the present study, increased expression and activity of antioxidative enzymes in the 4D-HBO_2_ group have been observed (Tables 4 and 5). The main source of superoxide in the 4D-HBO_2_ group seems to be NADPH oxidase. However, at the same time, there was a significant upregulation of SOD1 and SOD3 in the 4D-HBO_2_ group and a lower superoxide production observed in direct fluorescence measurements, speaking in favor of intermittent hyperoxygenation treatment as beneficial in increasing vascular antioxidative mechanisms. Similarly, some former studies reported that when HBO_2_ was administered for more than a single exposure, an adaptive mechanism which protects against further oxidative damage was activated [49, 50]. Thus, preconditioning with HBO_2_ treatment may be used to preserve several organs or tissues from following oxidative injuries [51, 52]. These adaptive and preconditioning actions triggered by HBO_2_ treatments may also be responsible for changes in the underlying mechanisms of vascular reactivity in the present and previous studies [8, 10, 53, 54]. Since HBO_2_ is an important therapy with life-saving properties in various conditions and its efficacy generally depends on repeated exposures for several days [55], it is of particular importance to define its molecular interactions when administered in a repetitive manner. The present results suggest that intermittent exposure to HBO_2_ leads to a more effective scavenging of ROS and to the activation of protective responses including antioxidant gene expression and increased antioxidative enzyme activities.

Even though unchanged activity of total SOD enzymes was surprising (Table 1), it is in concordance with the findings of Mamo et al., who related mRNA expression and activity of enzyme discordance with increased nitration of SOD [56]. It is known that hyperoxia increases ROS production, such as peroxynitrite. Reactive nitrogen species can inhibit activity of multiple antioxidants, including SOD [57]. Since SOD scavenges superoxide and prevents its reaction with NO, extracellular superoxide production in excess of the SOD antioxidant capacity promotes formation of both ROS and RNS [58]. This can occur due to loss of SOD activity or to increase in NO production [56, 59]. Another possibility involves enhanced generation of oxidants such as H_2_O_2_ under hyperoxic conditions. H_2_O_2_ has been shown to inactivate SOD through modification of the active site of the enzyme [60], and, for example, estimated brain H_2_O_2_ concentrations during HBO_2_ are as much as 2–7 times normoxic values [61]. Levels of H_2_O_2_ were not measured in this study, but increased mRNA expression and activity of CAT and GpX can support this explanation. Taken together, our results of increased mRNA expressions and no changes in activity of the SOD enzymes suggest efficient oxidative stress management in the 4D-HBO_2_ group who exhibited improved vasorelaxation.

Further studies concentrated on transcription factors, and their target genes known to be triggered and activated with HBO_2_ may help to elucidate the exact pathways and molecular interactions which occur during or after repeated HBO_2_ administrations [62].

## 5. Study Limitations

This study was done only on male rats to avoid any influence of sex hormones or sex itself. Previously, we did an aortic ring reactivity assay on female diabetic rats (data not published) where we observed a protective role of sex hormones [63]. Also it is known that oestrogenes can have antioxidant potential [64] and may improve redox balance and thus influence endothelium-dependent relaxation [65]; therefore, we choose to perform experiments on male rats only.

## 6. Conclusion

The results in this study showed impaired endothelial-dependent vasorelaxation in acute HBO_2_ which was transient and reversible and was caused by increased superoxide production and overall increased oxidative stress. On the other hand, intermittent HBO_2_ exposure exhibited a beneficial effect on vascular relaxation and preserved it, due to increased vascular antioxidant capacity. The present study is the first one to directly assess the production of superoxide and expression of antioxidative enzymes in different HBO_2_ protocols and to relate it to the vascular reactivity.

## Figures and Tables

**Table 1:** Relative expression of HPRT gene in aortic tissue.

	CTRL	A-HBO_2_	24H-HBO_2_	4D-HBO_2_
HPRT	305907953.65	582040046.02	206658013.40	17116739.21
	386556646.61	323880912.24	260830474.32	21126452.63
	259559691.14	466690666.09	281799045.13	24126211.32
	319452834.56	148010504.98	221585966.06	24391652.31
	447773644.68	162037010.83	352353682.25	59118846.34
	118147541.47	122675737.08	462065422.48	35533366.23

Average	306233052.02	300889146.21	297548767.27	30235544.67

**Table 2:** Measurements of body mass, arterial blood pressure, and mean arterial pressure.

	CTRL	A-HBO_2_	24H-HBO_2_	4D-HBO_2_
Body mass (g)	338.11 ± 8.66	336.33 ± 8.79	324.83 ± 12.48	320.71 ± 12.35
Systolic blood pressure (mmHg)	133.67 ± 1.28	98.33 ± 1.41^∗^	133.52 ± 2.95	136.47 ± 2.65
Diastolic blood pressure (mmHg)	93.65 ± 1.81	75.99 ± 3.65^∗^	96.12 ± 4.50	95.22 ± 3.43
Mean arterial pressure (mmHg)	106.99 ± 1.49	83.21 ± 2.39^∗^	108.59 ± 3.93	108.97 ± 2.81

Results are shown as mean ± SEM (standard error of mean); ^∗^*p* < 0.05 compared to the control group.

**Table 3:** The effects of hyperbaric oxygen on the level of oxidative stress (TBARS) and antioxidative capacity (FRAP) in serum samples.

	*N*	CTRL	A-HBO_2_	24H-HBO_2_	4D-HBO_2_
TBARS (*μ*mol l^−1^ MDA)	8	0.66 ± 0.02	0.86 ± 0.04^∗^	0.64 ± 0.02	0.62 ± 0.02
FRAP (mmol l^−1^ TROLOX)	8	0.17 ± 0.00	0.15 ± 0.01	0.17 ± 0.00	0.18 ± 0.00

Results are shown as mean ± SEM (standard error of mean); ^∗^*p* < 0.05 compared to the control group. TBARS: thiobarbituric acid-reactive substances; FRAP: ferric reducing ability of plasma, plasma antioxidant capacity.

**Table 4:** Relative expression of SOD1, 2, and 3, CAT, GPx1, GPx4, eNOS, iNOS, and NADPH oxidase component (p47phox and gp91phox) mRNAs in aortas.

Group	SOD1	SOD2	SOD3	CAT	GPx1	GPx4	eNOS	iNOS	p47phox	gp91x
CTRL	0.85 ± 0.15	0.43 ± 0.13	0.49 ± 0.15	0.42 ± 0.10	1.84 ± 0.65	0.35 ± 0.08	0.13 ± 0.06	0.38 ± 0.07	5.21 ± 1.22	0.10 ± 0.02
A-HBO_2_	0.82 ± 0.13	0.48 ± 0.16	0.46 ± 0.08	0.76 ± 0.18	3.51 ± 1.22	0.31 ± 0.11	0.03 ± 0.02	0.11 ± 0.02^∗^	3.87 ± 0.44	0.68 ± 0.21
24H-HBO_2_	0.86 ± 0.10	0.97 ± 0.23	0.82 ± 0.08	1.12 ± 0.18^∗^	2.72 ± 0.50^†^	0.63 ± 0.07^∗^^†^	0.13 ± 0.06	0.12 ± 0.03^∗^	4.44 ± 0.49	1.07 ± 0.11^∗^^†^
4D-HBO_2_	1.77 ± 0.20^∗^^†‡^	0.40 ± 0.15	1.49 ± 0.33^∗^^†‡^	1.66 ± 0.16^∗^^†‡^	10.86 ± 1.84^∗^^†‡^	0.48 ± 0.14^∗^^†^	0.04 ± 0.02	0.21 ± 0.05	6.96 ± 0.67^∗^^†‡^	1.95 ± 0.34^∗^^†‡^

Data are presented as mean ± SEM (standard error of mean); *n* = 6 (number of rats per group). SOD 1, 2, and 3: superoxide dismutase; CAT: catalase; GPx1 and 4: glutathione peroxidase; eNOS: endothelial nitric oxide synthase; iNOS: inducible nitric oxide synthase; p47phox and gp91phox: NADPH oxidase components. ^∗^*p* < 0.05 compared to the CTRL group; ^†^*p* < 0.05 compared to the A-HBO_2_ group; and ^‡^*p* < 0.05 compared to the 24H-HBO_2_ group.

**Table 5:** Antioxidant enzyme activities.

Experimental group	*N*	CAT (U/mg P)	GpX (U/mg P)	SOD (U/mg P)
CTRL	4	43.82 ± 7.34	0.16 ± 0.01	19.31 ± 0.34
A-HBO_2_	4	49.75 ± 2.58	0.14 ± 0.02	18.75 ± 0.32
24H-HBO_2_	4	57.80 ± 9.48	0.18 ± 0.02	18.89 ± 1.06
4D-HBO_2_	4	69.67 ± 4.54^∗^^†^	0.48 ± 0.10^∗^^†‡^	17.40 ± 0.25

Data are presented as mean ± SEM. ^∗^*p* < 0.05 compared to CTRL; ^†^*p* < 0.05 compared to A-HBO_2_; and ^‡^*p* < 0.05 compared to 24H-HBO_2_. P: protein; CAT: catalase; GpX: glutathione peroxidase; SOD: superoxide dismutase.
